# In Vitro and In Silico Interaction Studies with Red Wine Polyphenols against Different Proteins from Human Serum †

**DOI:** 10.3390/molecules26216686

**Published:** 2021-11-05

**Authors:** Raja Mohamed Beema Shafreen, Selvaraj Alagu Lakshmi, Shunmugiah Karutha Pandian, Young-Mo Kim, Joseph Deutsch, Elena Katrich, Shela Gorinstein

**Affiliations:** 1 Department of Biotechnology, Dr. Umayal Ramanathan College for Women, Algappapuram, Karaikudi 630003, India; beema.shafreen@gmail.com; 2 Department of Biotechnology, Alagappa University, Science Campus, Karaikudi 630003, India; lakshmivinay.317@gmail.com (S.A.L.); sk_pandian@rediffmail.com (S.K.P.); 3 Industry Academic Collaboration Foundation, Kwangju Women’s University, Gwangju 62396, Korea; bliss0816@kwu.ac.kr; 4 Institute for Drug Research, School of Pharmacy, Faculty of Medicine, The Hebrew University of Jerusalem, Jerusalem 9112001, Israel; josephd@ekmd.huji.ac.il (J.D.); ekatrich@gmail.com (E.K.)

**Keywords:** beverages, health properties, antioxidant activities, fibrinogen, albumin, rutin, tannic acid, resveratrol, binding properties

## Abstract

Previous reports have shown that consumption of wine has several health benefits; however, there are different types of wine. In the present study, red wines were investigated for their compositions of active ingredients. The interaction of each component in terms of its binding mode with different serum proteins was unraveled, and the components were implicated as drug candidates in clinical settings. Overall, the study indicates that red wines have a composition of flavonoids, non-flavonoids, and phenolic acids that can interact with the key regions of proteins to enhance their biological activity. Among them, rutin, resveratrol, and tannic acid have shown good binding affinity and possess beneficial properties that can enhance their role in clinical applications.

## 1. Introduction

Phenolic compounds are an essential part of the human diet and are of considerable interest due to their antioxidant properties and potential beneficial health effects. Their effects on human health depend on the amount consumed and on their bioavailability. Many studies have demonstrated that polyphenols also have good effects on the vascular system by lowering blood pressure, improving endothelial function, increasing antioxidant defenses, inhibiting platelet aggregation and low-density lipoprotein oxidation, and reducing inflammatory responses [1,2]; they are found in fruits [3], cereals, vegetables, legumes, chocolate, and beverages such as coffee, tea, beer, and wine. Polyphenols have been reported to inhibit platelet aggregation both in vitro and in vivo. The analysis of the results indicates a promising role for food polyphenols in preventing thrombosis and cardiovascular diseases, but, at the same time, suggests caution when transferring the in vitro findings in vivo [4,5,6]. It was reported in a number of recent studies [7,8,9,10] that the consumption of beer, wine, and fruits has already been associated with a multitude of beneficial effects due to their high polyphenolic content. Beverages have to be consumed moderately in order to obtain their positive influence on health. Only the high amount of bioactive compounds—and not the alcohol content—positively influences the human metabolism. The pro-oxidant effect of red wine polyphenols promotes an adaptive stress response in human erythrocytes, which enhances their antioxidant defense [11]. Phenolic compounds in wine, such as tannins, phenolic acids, and anthocyanins, are some of the determinants of its quality. It was shown that phenolic compounds participate not only in the appearance and sensory characteristics of wine, but also in its healthy properties [12]. As an example, research was conducted on 110 Italian red wines from a single vintage in order to determine the standard composition, color, and phenolic characteristics based on the parameters used in the wine industry [13]. The elevation of C-reactive protein (CRP) levels in blood was recognized as a cardiac disease risk factor. An increased nitration of fibrinogen has been reported in cardiovascular diseases. Consumption of wine is shown to reduce the risk of heart disease and improve longevity [14]. The defense mechanisms against nitrative modifications are crucial for the complex hemostasis process. Flavonoids have antioxidative properties and could protect biomolecules against the action of peroxynitrite [15]. There are reports that also show the results of phenolic compounds with the main human serum proteins [16]. Recently, research on wine has been widely cited, but in spite of a number of reports [17,18] and evidence that wine exerts beneficial effects on human health when it is consumed with moderation, how the main phenolics of red wine contribute to the quenching properties of the main human serum proteins has not been investigated. For this reason, red wine samples were investigated for their antioxidant activities, bioactive compounds, and interactions of wine polyphenols with the main serum proteins, with an emphasis on the influence of polyphenols and not the alcohol content on the health properties of wine. Fluorescence measurements, antioxidant assays, and docking analysis were applied for the first time to wine samples, and correlations were made among the results obtained in these studies.

## 2. Results

### 2.1. Bioactive Properties of the Investigated Wines

The results of the phenolic compounds are presented in Table 1.

Our results for the total polyphenols were in the ranges found for Italian red wines at 1065 mg GAE/L, with the average values ranging between 1945 and 2033 and 2841–3578 mg GAE/L; the total tannins were the lowest at 533 mg CE/L, with averages of 1341 and 2043 and a maximum of 2965 mg CE/L [13]. The polyphenols, flavonoids, and antioxidant activities were in the range of organic wines rather than the range of conventional ones. The results obtained were comparable to those of conventional red wines with regard to the total polyphenols, flavonoid content, phenolic profile, and antioxidant activity [5]. The total phenolic content and total flavanols of the wines were found to vary from 1439.8 mg to 2966.0 mg GAE/L and from 439.4 to 1367.7 mg CE/L, respectively, which was in line with the presented data (Table 1). The phenolic content values observed in the present study were similar to those reported by other authors for Cabernet Sauvignon wines, showing values from 1300 to 2900 mg GAE/L [19]. The final flavonoid content was 555 mg CE/L. These results were lower than those reported previously (1390 mg CE/L) [19]. The anthocyanin content was also lower than previously reported for Cabernet Sauvignon wines (between 300 and 320 mg C3G/L). The results of FRAP 5.08 mmol TE/L, ABTS 10.68 mmol TE/L, and DPPH 3.71 mmol TE/L were lower than those reported by other authors for red wines, as well as in comparison with the presented data (Table 1). The polyphenol content was 1268 mg GAE/L; the tannin content varied from 133.9 to 318.3 mg CE/L, and the anthocyanin content was observed to be 104.08 mg cyanidin 3-glucoside equivalent per liter (mg C3G/L) [20,21,22]. Our results were in line with others [23], where twenty seven wines—among them, twenty four were red wines and three were white wines—were analyzed using liquid chromatography and spectrophotometric analysis, and the amounts of total polyphenols of the red wines varied from the lowest sample of 1220 mg GAE/L to the highest one of 2413 mg GAE/L. The amounts of anthocyanins in the same samples [23] varied from the lowest of 79 to the highest of 232 mg malvidin-3, 5-diglucoside/L. For the red wines, the antioxidant capacities varied between 13.4 and 20 mmol/L [24,25].

### 2.2. Binding Properties of Wines and Some Phenolic Compounds with the Main Human Proteins

**Figure 1:**
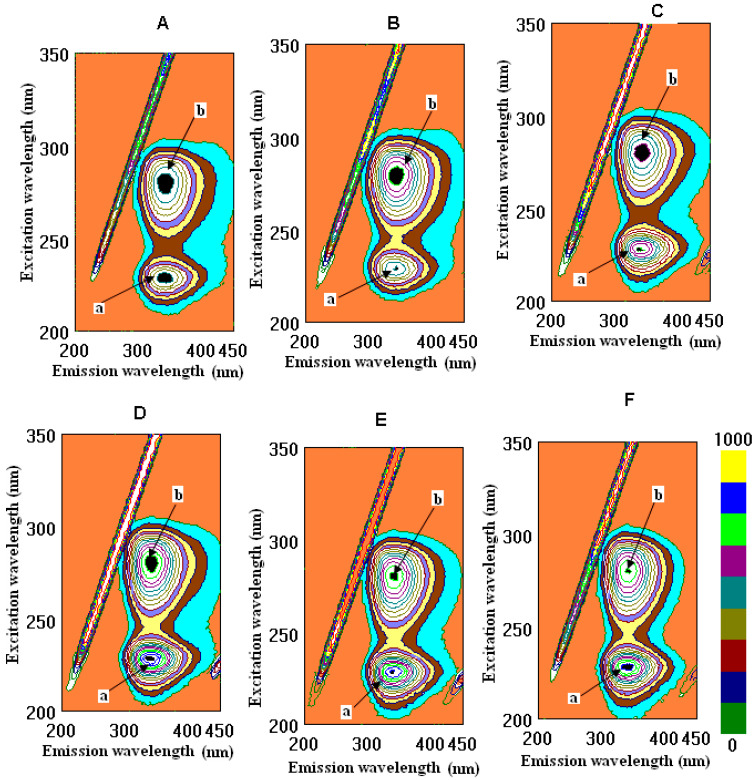
Fluorometric measurements in a three-dimensional fluorescence analysis (3D-FL) of red wine samples and standards after interaction with fibrinogen Cross-images of the results obtained from the 3D-FL of the investigated samples after interaction with fibrinogen are shown in the following order: (**A**) CSCarmel2; (**B**) CSYarden2; (**C**) tannic acid; (**D**) quercetin; (**E**) caffeic acid; (**F**) fibrinogen in solution. Abbreviations: CSCarmel2, Cabernet Sauvignon Carmel Selected, vintage 2019; CSYarden2, Cabernet Sauvignon Golan Heights Winery Yarden, vintage 2019. The locations of peaks **a** and **b** are shown in the figure and in Table 2 (for interpretation of the references to color in this figure legend, the reader is referred to the web version of this article).

**Figure 2:**
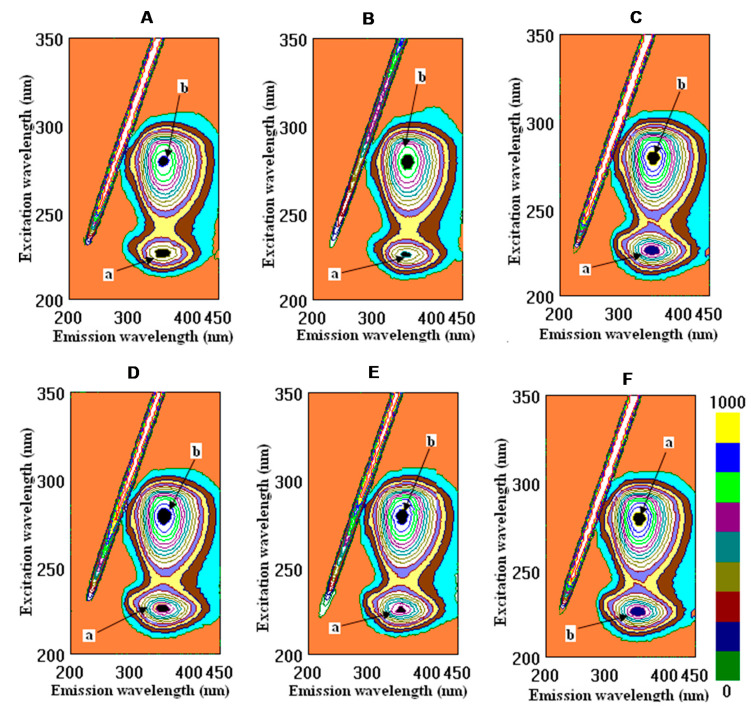
Fluorometric measurements in a three-dimensional fluorescence analysis (3D-FL) of red wine samples and standards after interactions with human serum albumin (HSA). Cross-images of the results obtained from the 3D-FL of the investigated samples after their interaction with HSA are shown in the following order: (**A**) CSCarmel2; (**B**) CSYarden2; (**C**) tannic acid; (**D**) quercetin; (**E**) caffeic acid; (**F**) HSA in solution. Abbreviations: CSCarmel2, Cabernet Sauvignon Carmel Selected, vintage 2019; CSYarden2, Cabernet Sauvignon Golan Heights Winery Yarden, vintage 2019. The locations of peaks **a** and **b** are shown in the figure and in Table 2 (for interpretation of the references to color in this figure legend, the reader is referred to the web version of this article).

The interactions of fibrinogen and human serum albumin with wine samples, tannic acid, quercetin, and caffeic acid in their cross-images are presented in Figure 1 and Figure 2.

The binding properties of wine samples and some individual phenolic compounds and standards were compared in terms of their interactions with native human serum albumin and plasma-circulating fibrinogen. Tannic and caffeic acids and quercetin were also compared with the natural fibrinogen.

Few studies of the roles played by different phenolic fractions in wine astringency using methods involving fluorescence spectra are available [26]. However, the polymeric proanthocyanidins were found to possess higher activity than the other fractions. This conclusion was corroborated by the findings in the spectral measurements of the fluorescence, which showed that the polymeric fractions exhibited a significantly stronger quenching of the fluorescence of bovine serum albumin (BSA). The fluorescence intensity of BSA in the absence of samples at the emission maximum (335 nm) was approximately 541.68. It was observed that, for BSA, there was a noticeable decrease in the fluorescence intensity upon binding to polymeric fractions. In the present study, at the emission maximum (228 nm), HSA was approximately 643.0. As was shown, CSYarden2 exhibited a significantly stronger function of binding for the fluorescence of HSA than in the other samples, except for tannic acid, based on the calculations of the intensity of peak **b** (Figure 2, Table 2). Among these investigated samples, the intensity decreased for peak **a** in the order ethanol > caffeic acid > quercetin > tannic acid > CSYarden2 > CSCarmel2 > CSYarden1 > CSCarmel1, which means that CSCarmel1 possessed a higher affinity for HSA than the other samples did. For peak **b**, a slight change in the order appeared: ethanol > quercetin > caffeic acid > CSCarmel2 > CSCarmel1 > tannic acid > CSYarden2 > CSYarden1. These results are in agreement with the data on BSA and its interactions with polymeric fractions that were also found in wines [27]. Recently, the interactions of flavonoid–metal complexes with serum albumin (SA) have been widely studied because the complexation has a significant impact on biological activities [28]. The strength of binding might be increased in the presence of Cu (II), as evidenced by the calculation of the binding constant. However, the drug binding sites in BSA and HSA are not altered during the complexation process. Both HSA and BSA exhibit similar kinds of fluorescence emissions based on excitation at 295 nm due to the presence of tryptophan residue (Trp 214 in HSA and Trp 134/Trp 213 in BSA). The fluorescence of BSA/HSA was observed to be quenched in the presence of both quercetin and its Cu (II) complex [28]. The fluorescence intensity of BSA decreased with an increase in flavonoid concentration in the model wine with or without ultrasonic irradiation, which is in line with the data shown in Table 2. A characteristic fluorescence emission spectrum of BSA is displayed with a maximum value at the wavelength of 336 nm, which is mainly attributed to the tryptophan (Trp) residues [17]. The data on the fluorescence measurements of the interaction of tannic and gallic acids with HSA (Table 2) are comparable with the data on the interactions of a typical gallotannin 1,2,3,4,6-penta-O-galloyl-β-D-glucopyranose (PGG) and two simple phenolic compounds, ellagic acid (EA) and gallic acid (GA), with BSA. Fluorescence experiments showed that PGG and EA could strongly interact with BSA. The binding constants showed a pH-dependent binding of phenolic acids by BSA. PGG has a greater impact on the secondary structure of BSA when compared with small molecular compounds GA and EA [29]—which is also typical during the interaction of wine polyphenols with tannic acid—in comparison with gallic acid. The interaction between polyphenols and HSA/BSA is widely used not only for pharmaceutical applications, but also as an index of the quality of red wines. The skin and seed extracts of grapes were analyzed in terms of their fluorescence spectra. The fluorescence emission spectra of BSA with three kinds of grape varieties—Cabernet Sauvignon, Merlot, and Cabernet Gernischet—were obtained upon the addition of the skin and seed extracts; the fluorescence quenching of BSA in the presence of extracts from grape skins and seeds was evaluated by using fluorescence spectrometry based on simulated conditions of human physiological conditions (pH = 7.4) in order to study the phenolics. A decrease in the fluorescence intensity caused by the quenching of the skin and seed extracts was observed, which is in line with the action of red wine polyphenols [30]. The presence of phenolic compounds can make a great contribution to the perception of astringency in red wines based on their interactions with proteins. Human salivary protein and bovine serum albumin were used in this study to investigate the relationship between astringency and polyphenol composition. The results indicated that a positive correlation existed between the percentage of polymeric proanthocyanidins and the total phenols. In comparison with other fractions, the polymeric fractions exhibited the highest affinity for proteins and, thus, the highest astringency [27]. The results of previous reports revealed that both original and lyophilized wines exercise statistically significant beneficial lipidemic and antioxidant effects by reducing total cholesterol (TC), low-density lipoprotein cholesterol, triglycerides, and lipid peroxides, as well as by elevating the high-density lipoprotein cholesterol–TC ratio. There were no statistically significant differences in the results between groups that were fed a basic diet (BD) supplemented with original wine versus groups fed BD supplemented with lyophilized wine. Therefore, it can be concluded that the biologically active compound of this beverage is the dry matter containing, inter alia, polyphenols in relatively high concentrations [4,31,32]. This conclusion is also in agreement with the present results, which show very low values of binding with ethanol (Table 2).

### 2.3. Docking Studies

As a complex mixture, red wine has been shown to have several health benefits in the improvement of cardiovascular disease and cancer, as well as in its antioxidant and anti-inflammatory properties. Red wine as such has been defined according to its antioxidant role; however, the functional role of each molecule has not been explored. Hence, in the present study, interactions of flavonoids (epicatechin, epigallocatechin, quercetin, rutin, and myricetin), non-flavonoids (resveratrol), and phenolic acids (caffeic, gallic, and tannic acids) were investigated with respect to different proteins from human serum (CRP, fibrinogen, GPX3, and HSA). From the interaction study, it was observed that rutin, a flavonoid, had the highest binding affinity with the serum proteins (Table 3).

However, resveratrol showed a similar binding affinity to that of flavonoids, though its presence in the red wine was observed to be very low compared to the other polyphenols. Among the phenolic acids, tannic acid exhibited the highest binding affinity with the target proteins in comparison with gallic and caffeic acids.

#### 2.3.1. Interaction Analysis with C-Reactive Protein (CRP)

**Figure 3:**
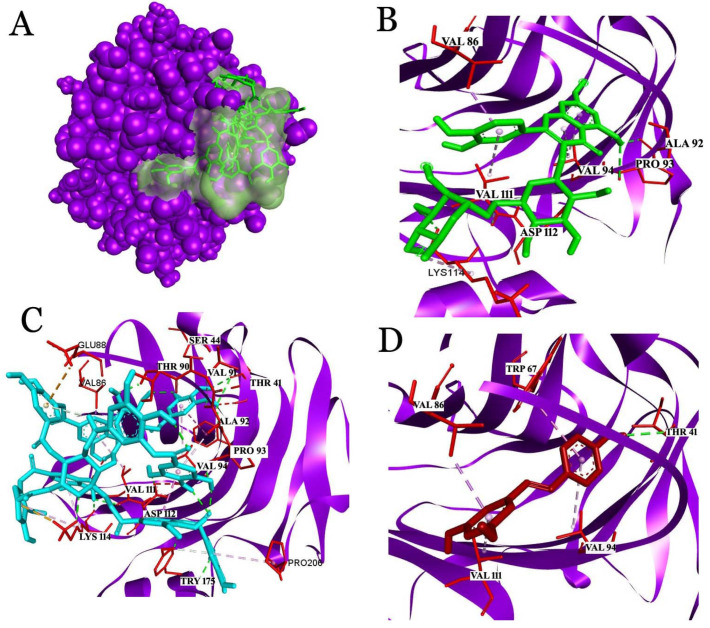
Interactions of ligands with the key residues of C-reactive protein (CRP). (**A**) The surface view represents the predicted binding pocket (green color) of the CRP; (**B**) the interaction of rutin with CRP; (**C**) the interaction of tannic acid with CRP; (**D**) the interaction of resveratrol with CRP.

The molecular docking of rutin with CRP showed a binding affinity of −8.7 kcal/mol and interactions with Val86, Ala92, Pro93, Val94, Val111, Asp112, and Lys114 (Figure 3). Among the amino acids, Asp112 and Lys114 played critical roles in the formation of the C1q binding with CRP. C1q, as the first subcomponent of the classical pathway, initiates an anti-inflammatory response in association with CRP and the phagocytosis of apoptotic cells [33]. Rutin showed a covalent interaction with Asp112 and a hydrogen interaction with Lys114, which supports the stable interaction of the ligand with the receptor. Similarly, with a binding affinity of −7.4 kcal/mol, resveratrol showed interactions with Thr41, Trp67, Val86, Val94, and Val111. Resveratrol has been established to have a hydrogen bond interaction with Thr41. The regions of CRP covering from 35 to 47 amino acids are considered to be important residues for therapeutical and diagnostic studies. Thus, the interaction of resveratrol with Thr41 from CRP is considered significant for clinical studies. Tannic acid, a phenolic acid that was used for this study, showed a binding affinity of −7.7 kcal/mol and interactions with several critical residues: Asp112, Lys114, and Tyr 175 of CRP.

#### 2.3.2. Interaction Analysis with Fibrinogen

**Figure 4:**
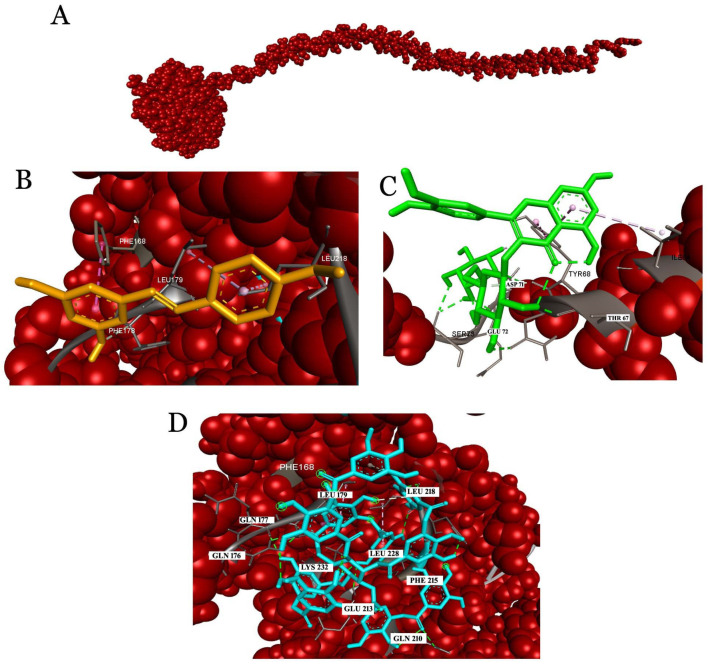
Molecular docking of ligands with fibrinogen. (**A**) Fibrinogen represented as a CPK model; expanded view of the ligands showing their interactions with the critical residues of fibrinogen; (**B**) rutin; (**C**) tannic acid; (**D**) resveratrol.

The docking of fibrinogen with wine compounds revealed rutin as the top scorer with the highest binding affinity of −7.9 kcal/mol, followed by tannic acid with a binding affinity of −6.4 kcal/mol. The amino acids from the gamma chain involved in the interactions were Phe168, Gln176, Gln177, Phe178, Leu179, Arg197, Asp199, Gly200, Val202, Asp203, Phe204, Lys205, Glu210, Phe215, His217, Leu218, Glu225, Leu228, Lys232, Gly331, Asn345, Gly346, Tyr349, Val347, Tyr348, Gln350, Gly351, Thr353, Tyr354, Ser358, and Pro360 (Figure 4). The D-fragment of the fibrinogen gamma chain (γC domain) acted as a ligand and interacted with leukocyte integrin αmβ2 (CD11b/CD18, also known as Mac-1 or CR3). In particular, two peptides, P2 (γ377–395) and P1 (γ190–202), have been reported to be implicated in binding integrin αmβ2. Though P2 and P1 were demonstrated to be binding sites for integrin αmβ2, P2 was reported to exhibit high binding affinity to the αmI domain of integrin αmβ2. The binding of fibrinogen binding to leukocytes’ integrin recruits the phagocyte during inflammatory response [34,35]. In addition, the pleiotropic role of fibrinogen in neurological diseases has been described [36,37]. Intriguingly, our docking analysis of fibrinogen with components of red wine unveiled P1 as the binding site of flavonoids and phenolic acids.

#### 2.3.3. Interaction Analysis with Human Glutathione Peroxidase 3 (GPX3)

**Figure 5:**
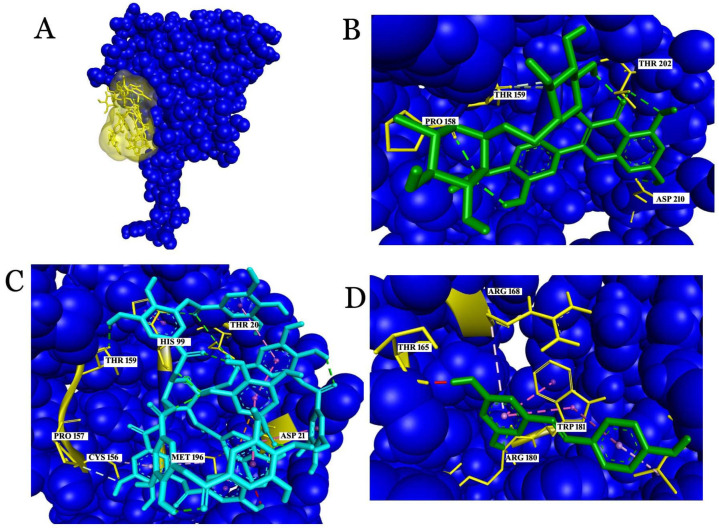
In silico docking of ligands into the binding pocket of human glutathione peroxidase 3 (GPX3). (**A**) CPK model of GPX3 protein, in which the yellow-colored surface represents the binding pocket; the expanded views of ligands interacting with the key amino acids of GPX3 (CPK model); (**B**) rutin; (**C**) tannic acid; (**D**) resveratrol.

Similarly, in GPX3, rutin had the highest binding affinity of −7.4 kcal/mol, and the second top scorer, tannic acid, had a binding affinity of −7.3 kcal/mol; the amino acids that made non-covalent interactions were Thr20, Asp21, Tyr43, Gly44, Ala45, Leu46, Tyr53, His99, Phe135, Gln136, Lys137, Gly138, Asp139, Lys144, Glu145, Gln146, Lys147, Cys156, Pro157, Thr159, and Met196 (Figure 5). GPX3, an antioxidant enzyme in plasma proteins, contains Sec73 as an active site. Though the docking analysis revealed a high binding affinity for rutin and tannic acid, the non-flavonoid resveratrol showed an interaction at the active-site pocket residues of Sec73 [38,39]. Apart from this, residues from the pocket interface, such as Gly74, Leu75, Arg180, Trp181, His200, and Arg201, were implicated in the interactions.

#### 2.3.4. Analysis of the Interaction with Human Serum Albumin (HSA)

**Figure 6:**
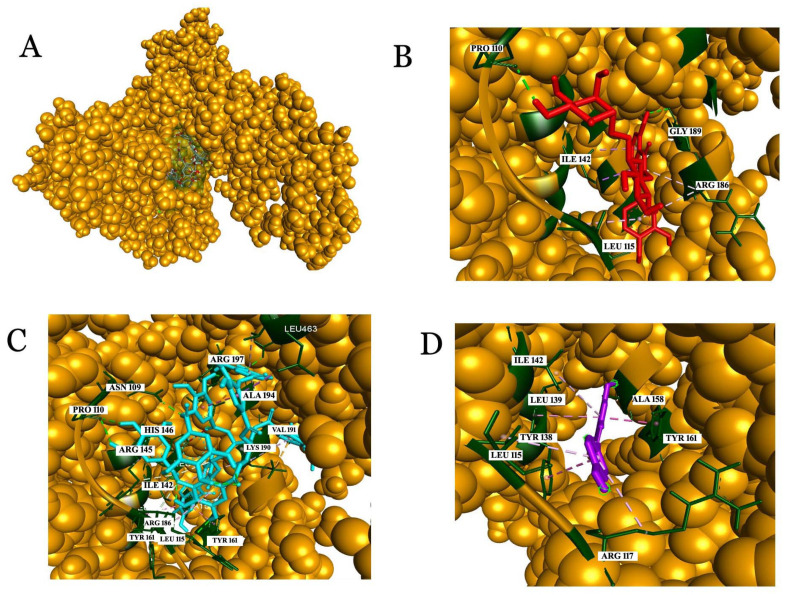
Analysis of interactions with human serum albumin (HSA). (**A**) The green-colored surface represents the binding pocket of the HSA protein (CPK model). (**B**) Interaction of rutin with the amino acids in the binding pocket, (**C**) tannic acid, and (**D**) resveratrol.

In the case of HSA, tannic acid exhibited the highest binding affinity of −10.4 kcal/mol, followed by rutin with a binding affinity of −9.9 kcal/mol. The binding was driven by Asn109, Pro110, Asn111, Leu112, Arg114, Leu115, Arg117, Pro118, Met123, Ile142, Arg145, His146, Phe149, Leu154, Phe157, Tyr161, Leu182, Arg186, Gly189, Lys190, Val191, Ser193, Ala194, Arg197, and Leu 463 (Figure 6). Almost all of the compounds from wine used for the study were shown to have interactions with HSA. Interestingly, HSA is an important protein in plasma, which acts as a drug carrier and in the transportation of key enzymes, fatty acids, and biomolecules in the circulatory system. Thus, the interactions of the active ingredients in wine with HSA can be used in mainstream albumin fusion technology or albumin-based encapsulation methods for developing drug molecules for clinical applications.

## 3. Discussion

Overall, tannic acid has been reported to interact with human plasma proteins, and our results are in good agreement with previous reports [40]. Hence, it is not surprising to observe the high binding affinity between tannic acids and plasma proteins, such as HSA, GPX3, and fibrinogen. Moreover, phenolic acids have been reported to interact with plasma proteins through non-covalent interactions, such as hydrogen bonds and electrostatic, van der Waals, or hydrophobic interactions [41]. Our docking analysis also showed the possible interactions, such as hydrogen bonds, van der Waals interactions, and electrostatic interactions, with the target proteins. Tannic acid has been reported to lower blood pressure in hypertensive rats [42], to have a protective role against acute doxorubicin-induced cardiotoxicity in Sprague–Dawley rats [43], to lower the malondialdehyde (MDA) level (a marker of oxidative stress), and to increase the antioxidant enzymes, such as superoxide dismutase, glutathione peroxidase, and catalase, thereby enhancing the antioxidant properties. In addition, anticancer [44], neuroprotective [45,46], anti-neuro-inflammatory [47], anti-inflammatory [48], antimicrobial, and antibiofilm activity [49,50] has been reported. On the contrary, tannic acids are mainly used in topical applications due to their bioavailability after ingestion, high molecular weight (>1000), low lipid solubility, and high affinity to bound to plasma proteins. In addition, their mode of elimination from the human system is still unclear. Rutin, a flavonoid, showed the highest binding affinity with all target proteins (CRP, fibrinogen, GPX3, and HSA) in our present study.

Rutin has also been investigated due to its beneficial effects on the strengthening of blood vessels and arteries [51], as well during the chronic condition of arthritis, as it can ease the flexibility of the veins [52]. Rutin has also been used for several pharmacological applications, including as a model drug, due to its antifungal, antiviral, and anti-inflammatory properties [53,54,55]. The cytotoxicity of heme and oxidative stress in endothelial cells were decreased in the BSA–diligand complexes relative to those of heme or BSA–heme complexes, and the co-presence of rutin played an important role. These results suggest the possibility and advantage of developing BSA-based carriers for the suppression of heme toxicity in biomedical applications [56]. Apart from this, rutin has also been applied in industry as a colorant, stabilizer, and preservative for food [57]. However, as an important common dietary flavonoid, studies of rutin have been focused on its formulation and bioavailability [58]. Thus rutin, was found to be a promising nutraceutical for the prevention and treatment of chronic human diseases according to hematological, biochemical, and histological evidence and due to the drug delivery [59,60]. However, rutin is the compound with the lowest molecular weight (610.5 g/mol) among the polyphenols, and therefore, it is regarded as significant for clinical applications. Interestingly, the non-flavonoid compound resveratrol was identified in red wine, though its presence was very low. Among other compounds, resveratrol has numerous biological activities. Resveratrol has shown antiviral activity against several viruses, such as polyomavirus, influenza virus, respiratory syncytial virus, MERS-CoV, and the emerging SARS-CoV [61]. The mechanisms of action of three important compounds (resveratrol, rapamycin, and metformin) on the cellular pathways involved in viral replication and the mechanisms of virus-related diseases, as well as the current status of their clinical use, were discussed [62]. The antiviral effect of resveratrol against MERS-CoV was evaluated through antiapoptotic and viral titer assays. The results revealed that resveratrol targeted caspase 3 in Vero E6 cells, as well as the nucleocapsid protein of MERS CoV. A concentration ranging from 31.5 to 125 μM and 0.5 mg/mL (2.05 mM) was shown to have a positive effect against MERS-CoV and SARS-CoV, respectively, in Vero E6 cells [63,64]. Moreover, it reduced the viral RNA levels and infectious titers. In the case of SARS-CoV, resveratrol derivatives suppressed the replication of SARS-CoV and decreased the cytopathic effects [63]. Resveratrol was demonstrated to be a stimulator of fetal hemoglobin and a potent antioxidant by trapping reactive oxygen species. Resveratrol could be proposed as potential therapeutic in the treatment of SARS-CoV-2 [65]. Resveratrol has been reported for its excellent pharmacokinetic characteristics, which have marked it for use as a potential drug candidate. When ingested, resveratrol can be easily absorbed in the gastrointestinal tract and rapidly metabolized in the liver into sulfate and glucuronide conjugates. Later, the metabolites are easily excreted through urine [63]. Thus, with its good absorption, low molecular weight (228.24 g/mol), low bioavailability, and extensive metabolism and excretion, resveratrol is considered to be a suitable drug candidate for oral administration during clinical ailments. To summarize the presented information about polyphenols and flavonoids in red wines, their high affinity and improvement of pharmacological actions, it is important to verify the exact mechanisms of their action. As discussed, polyphenols are among the molecules with pharmacological activity produced by plants. As drugs, these, due to their molecular structure, also have the ability to interact with molecules in our body, presenting various pharmacological properties [66]. Tannic acid and flavonoids play a role in the prevention of some diseases, and specifically in atherosclerosis. As was shown above, tannic acid is present in varying concentrations in plant foods and in relatively high concentrations in green teas and red wines. Human ether-a-go-go-related gene (hERG) channels are expressed in multiple tissues and play important roles in modulating the potential repolarization of cardiac action. Lung Ching green tea and red wine inhibited hERG currents with IC50 values of 0.04% and 0.19%, respectively. The effects of tannic acid, teas, and red wine on hERG currents were found to be irreversible. These results suggest that tannic acid is a novel hERG channel blocker, and consequently, it provides new mechanistic evidence for the understanding of the effects of tannic acid and the potential pharmacological basis of tea-and red-wine-induced biological activity [67]. As previously discussed, resveratrol first came to our attention in 1992, following reports of the cardioprotective effects of red wine. Thereafter, resveratrol was shown to exert antioxidant, anti-inflammatory, anti-proliferative, and angio-regulatory effects against atherosclerosis, ischemia, and cardiomyopathy. Although resveratrol has been claimed to be a master anti-aging agent that is effective against several age-associated diseases, a further detailed mechanistic investigation is still required in order to thoroughly unravel the therapeutic value of resveratrol against cardiovascular diseases at different stages of disease development [68]. Rutin and hesperidin were investigated in vitro for their anticoagulant activity through coagulation tests in terms of activated partial thromboplastin time (aPTT), prothrombin time (PT), and thrombin time (TT). Only an ethanolic solution of rutin at the concentration of 830 µM prolonged the aPTT, while the TT and PT were unaffected [69]. According to the results obtained and based on numerous recent reports [7,66,67,68,69], it was declared that wines possess a high bioactivity that allows them to be settled in the industries of food additives and medicinal products. In addition to phenolics, a number of other compounds are responsible for the quality of wine, as they determine the overall organoleptic impression of the wine in addition to the content of phenolic compounds, which are also present in large amounts in many other food products. Very recent publications mostly cited the results of phenolic compounds as secondary metabolites that are known to play crucial roles in important chemical reactions that impact the mouthfeel, color, and aging potential of red wine [70]. The phenolic profile—particularly the tannin concentration and structure—was the most important predictor of astringency and its subcomponents [71]. There were attempts to fingerprint the quality of red wines by using the ratio of the polymeric pigments and malvidin-3-glycoside, which was demonstrated to be a promising and practical chemical parameter for the rapid and simple assessment of the age of dry red wines. [72]. Volatile substances also play an important role in the quality of red wines, and it was found that 2-phenylethyl acetate, ethyl nonanoate, 2-hexanol, isoamyl octanoate, and ethyl 2-hydroxymethylbutanoate were the primary compounds responsible for wine classification [73]. The effects of various wine polyphenolic compounds were evaluated; among the wine phenolics tested, quercetin and resveratrol, in a dose-dependent manner, suppressed cytokine-induced C-reactive protein expression. Wine phenolics inhibited CRP expression [14]. Oversimplifications of the above results were indicated in reports [2,7,14] that the consumption of antioxidant-/polyphenol-rich foods might, therefore, impart anti-thrombotic- and cardiovascular-protective effects via their inhibition of platelet hyperactivation or aggregation. As a result of the ability of polyphenols to target additional pathways of platelet activation, they may have the potential to substitute or complement currently used anti-platelet drugs in sedentary, obese, pre-diabetic, or diabetic populations who can be resistant or sensitive to pharmacological anti-platelet therapy [2]. The health-promoting effects of red wine have been supported by epidemiological evidence, indicating that its components could improve endothelial dysfunction and hypertension, dyslipidemia, and metabolic disorders. The positive role of red wine in human health has been attributed to its phytochemical compounds, including polyphenols, as suggested by several clinical trials, including our previous studies in vivo [8]. The alcohol content of the investigated wines was 14.5%. The fluorescence measurements and calculations showed a significant difference between the original wine samples, tannic acid, and ethanol that were investigated in their interactions with fibrinogen and HSA. The binding properties of the tannic acid and wine samples were about 10–20 times higher than with those of ethanol, showing that the dry matter of the samples had higher bioactivity than the samples containing alcohol (Table 2). The biological role of red wine polymers remains largely unknown, although in vivo and in vitro antioxidant activity has been shown, which contributes against oxidative stress. The biological properties of red wine are therefore explained by the presence of phenolic compounds that are able to interact with physiological targets. The potential cardioprotective activity of red wine polyphenols has mainly been linked to the inhibition of platelet aggregation.

## 4. Materials and Methods

### 4.1. Reagents and Chemicals

Caffeic and gallic acids, catechin, epicatechin, quercetin, Trolox, Folin-Ciocalteu reagent, human serum albumin, fibrinogen, sodium nitrite, iron (III) chloride hexahydrate, 2,2-diphenyl-1-picrylhydrazyl (DPPH) and 2, 4, 6-tripyridyl-s-triazine (TPTZ) aluminum chloride, potassium peroxodisulfate and 2,2-azino-bis(3-ethylbenzothiazoline-6-sulfonic acid) diammonium salt (ABTS), copper (II) chloride dihydrate, sodium hydroxide, hydrochloric acid (37% *w*/*w*), 2,9-dimethyl-1,10-phenanthroline (neocuproine), and glacial acetic acid were obtained from Sigma (St. Louis, MO, USA). Standard phenolics were dissolved in methanol (1 mg/mL) and stored at −80 °C. All reagents and chemicals were of analytical grade.

### 4.2. Samples

Commercial wine bottles were purchased at wine shops and were investigated in this study. Every sample was bought in five bottles from wine shops in different locations; each had the same vintage, was from the same wine company and the same batch, and had an identical shelf life. The samples with a range of alcohol in same volume and from the same bottle were frozen at −80 °C to assess their antioxidant status and bioactivity. Cabernet Sauvignon wine samples of 2017 and 2019 vintages were purchased: Carmel Selected Cabernet Sauvignon 2017 (CSCarmel1) and 2019 (CSCarmel2) with 14.5% alcohol and Golan Heights Winery Yarden Cabernet Sauvignon 2017 (CSYarden1) and 2019 (CSYarden2) with 14.5% alcohol. The wines were transferred into 50 mL conical centrifuge tubes and stored at 8 °C. Samples were taken out of the refrigerator prior to, yesanalysis and were diluted according to the methods explained here.

### 4.3. Analyses of Bioactive Compounds

The total phenolic content (TPC) was measured by using the Folin–Ciocalteu method [74]. In brief, 250 μL of wine was mixed with 1000 μL of sodium carbonate (7.5%) and 1250 μL of Folin–Ciocalteu’s (10% in water) reagent. The mixture was incubated for 15 min at 50 °C in the dark (water bath) and measured at 765 nm using a spectrophotometer (Hewlett-Packard, model 8452A, Rockvile, MD, USA). Gallic acid was used as the standard, and the results were expressed as milligrams of gallic acid equivalent per liter (mg GAE/L).

The total flavonoid content (TFC) in the wine was measured by following the AlCl_3_ complexation method. Briefly, 31 μL of the sample was mixed in a microplate with 125 μL of water, 9.3 μL of sodium nitrate (5%), 9.3 μL of aluminium chloride (10%), and 125 μL of sodium hydroxide (0.5 M). The mixture was incubated for 30 min at room temperature in the dark. The reaction was measured at 510 nm. Catechin was used as the standard [75], and the results were expressed as milligrams of catechin equivalent per liter (mg CE/L).

The total flavanol content was determined [76] by measuring at 640 nm using 0.1 mL of the wine sample and 3 mL of 0.1% p-dimethylaminocinnamaldehyde (DMACA) solution (0.1% in 1 mol/L HCl in methanol). The results were expressed as catechin equivalent (CE).

The total tannins (TNs) were estimated by using spectrophotometric measurements of 0.5 mL of wine, where 3 mL of a 4% methanol vanillin solution and 1.5 mL of concentrated hydrochloric acid were added. The mixture was allowed to stand for 15 min. The absorption of the samples and a blank against water was measured at 500 nm [77].

The anthocyanin content (AC) in the wines was measured according to the following method [20,78]. An aliquot of 250 μL of the wine sample was poured into a tube with 2 mL of potassium chloride solution (0.025 M) and then adjusted to pH 1 with concentrated HCl. The mixture was incubated at room temperature for 20 min. In another tube, 250 μL of wine was mixed with 2 mL of sodium acetate solution (0.4 M, pH 4.5) and incubated at room temperature for 20 min. The absorbance of an aliquot of 300 μL of each sample was measured at 520 and 700 nm. The anthocyanin content in the samples was calculated in the following way: mg C3G/L = A × MW × D.F. × 103/ε × 1.

Here, A = (Abs520−Abs700), pH 1−(Abs520−Abs700) pH 4.5, MW (molecular weight) = 449.2 g/mol− for cyaniding 3-glucoside, D.F. = the dilution factor used, 103 = factor conversion of g to mg, ε = 26,900: molar extinction coefficient in L/mol cm, and 1 = the path length in cm. The results were expressed as milligrams of cyanidin 3-glucoside equivalent per liter (mg C3G/L).

The 2, 2′-azino-bis (3-ethyl-benzothiazoline-6-sulfonic acid) diammonium salt (ABTS) radical cation was formed by the ABTS solution (7 mM) with potassium persulfate (2.45 mM) in distilled water at room temperature at 16 h before use. A working solution (ABTS reagent) was diluted to obtain absorbance values of 0.7 at 734 nm and equilibrated at 30 °C. After the addition of ABTS solution, the absorbance reading was taken 1 min after the initial mixing and for up to 6 min; the percentage inhibition of absorbance was calculated with reference to a Trolox calibration curve and evaluated as mM Trolox equivalent/L of wine [20,79].

The antioxidant capacity in the wines was measured by using FRAP in 24 μL of the sample, which was mixed with 180 μL of FRAP reagent (TPTZ 10 mM in HCl 40 mM, iron chloride hexahydrate 20 mM, acetate buffer 0.3 M, pH 3 in a ratio of 1:1:10, prepared daily). The reaction was carried out at 37 °C, and the absorbance was measured at 595 nm every min for 30 min [80].

The antioxidant capacity was measured byusing 1, 1-diphenyl-2-picrylhydrazyl (DPPH) in 25 μL of the sample, which was mixed with 180 μL of DPPH radical at 6 mM and measured at 517 nm every 30 s for 10 min. Trolox was used as a standard for all antioxidant methods, and the results were expressed as millimoles of Trolox equivalent per liter (mmol TE/L) [20,81].

For the Cupric-Reducing Antioxidant Capacity (CUPRAC) assay [82,83], the red wines were diluted in a ratio of 1:10 (*v*/*v*) with dH_2_O. Prior to the determination, 1.0 mL of each of three solutions containing 0.010 M Cu (II), ammonium acetate buffer at pH 7.0, and 0.0075 M neocuproine (2,9-dimethyl-1,10-phenanthroline) in EtOH was mixed with 0.5 mL of the appropriately diluted sample together with 0.6 mL of dH_2_O in a tube. The reaction mixture was left for 1 h in the dark, and then the absorption was measured at 450 nm.

Some bioactive compounds, such as rutin, resveratrol, quercetin, caffeic acid, catechin, and epicatechin, were determined with an HPLC system [84,85,86]. A volume of 50 mL of each of the wine samples was extracted three times with 25 mL of diethyl ether and then three times with 25 mL of diethyl acetate, and the organic fractions were combined. After 30 min of drying with anhydrous Na_2_SO_4_, the extract was filtered through a Whatman-40 filter and evaporated to dryness in a rotary evaporator. The residue was dissolved in 2 mL of methanol/water (1:1, *v*/*v*) and analyzed by using high-performance liquid chromatography (HPLC). A Waters (Milford, MA, USA) chromatograph equipped with a 600-MS controller, a 717 plus autosampler, and a 996 photodiode-array detector was used. A gradient of solvent A (water/acetic acid, 98:2, *v*/*v*) and solvent B (water/acetonitrile/acetic acid, 78:20:2, *v*/*v*/*v*) was applied to a reverse-phase Nova-pack C18 column (30 cm × 3.9 mm internal diameter (I. D.) as follows: 0–55 min, 80% B linear, 1.1 mL/min; 55–57 min, 90% B linear, 1.2 mL/min; 57–70 min, 90% B isocratic, 1.2 mL/min; 70–80 min, 95% B linear, 1.2 mL/min; 80–90 min, 100% B linear, 1.2 mL/min; 90–120 min. For the HPLC analysis, an aliquot (50 µL) was injected into the column and eluted at the temperature of 20 °C. The samples were prepared and analyzed.

### 4.4. Fluorometric Studies

The profiles and properties of the polyphenols in wine were determined by using the two- (2D-FL) and three-dimensional (3D-FL) fluorescence (model FP-6500, Jasco spectrofluorometer, serial N261332, Tokyo, Japan). The 2D-FL measurements were taken at emission wavelengths from 310 to 500 nm and at an excitation of 295 nm. The 3D-FL was measured at emission wavelengths between 200 and 795 nm, and the initial excitation wavelength was 200 nm. For comparison of the obtained results, caffeic acid, quercetin, and tannic acid were used [9,10]. The binding properties of wine to human serum albumin (HSAlb) and fibrinogen were evaluated by using 2D- and 3D-FL. For the fluorescence measurements, 3.0 mL of 1.0 × 10^−5^ mol/L HSA was prepared in 0.05 mol/L Tris–HCl buffer (pH 7.4) containing 0.1 mol/L NaCl. Fibrinogen stock solution was made by dissolving in phosphate buffer (10 mM, pH 7.4) to obtain a concentration of 20 µM. Standard phenolic solutions, such as tannic acid, quercetin, and caffeic acid stock solution, were prepared daily by dissolving at a concentration of 10 mM in methanol and then diluting with 10 mM phosphate buffer at pH 7.4. All samples were kept at 4 °C before the analysis. The initial fluorescence intensities of albumin and fibrinogen were measured before their interactions with the investigated samples, as pure substances, and after their interactions with the samples (quenching of fluorescence emissions of proteins—in our case, albumin, fibrinogen, and polyphenols of wines). As mentioned above, the changes in the fluorescence intensities were used in the estimation of the binding activities [87].

### 4.5. Molecular Docking of Ligands with the Serum Proteins

Molecular docking studies of the docking of the compounds identified in red wine into serum protein targets were carried out by using AutoDock Vina [88]. For the studies, receptor proteins, such as human C-reactive protein (CRP) (PDB ID: 1B09), human serum albumin (HSA) (PDB ID: 1H9Z), human glutathione peroxidase 3 (GPX3) (PDB ID: 2R37), and human fibrinogen (PDB ID: 3GHG)—with resolutions of 2.5, 2.5, 1.85, and 2.9 Å, respectively—were obtained in pdb format from the PDB database. The crystal structures were pre-processed through the removal of all water and the addition of Kollman charges. The Gasteiger charges and hydrogen bond optimization was performed with AutoDock MGL tools version 1.5.6. Chain A of the grid-box of the receptor proteins CRP (144 Å × 157 Å × 27 Å), GPX3 (25 Å × 43 Å × 37 Å), and HSA (87 Å × 51 Å × 86 Å) was generated by AutoGrid4. For fibrinogen (213 Å × 55 Å × 87 Å), the gamma chain was selected for the study. The outputs of the best ligands and their interactions in the the molecular docking were analyzed through the BIOVIA-DS 17 R2 client (Dassault Systèmes Biovia Corp^®^, San Diego, CA, USA).

### 4.6. Data Analysis

All data obtained were calculated on the basis of a statistical analysis of Duncan’s multiple range test. Values were means ± SD per liter of 25 measurements, representing the commercial status of the wines and their replicates. Five replications of five wine samples from each vintage were used. To determine the statistical significance at the 95% interval of reliability, one-way analysis of variance (ANOVA) was used.

## 5. Conclusions

Previous reports have shown that the consumption of wine has several health benefits. However, there are different types of wine; in the present study, red wine was investigated for its composition of active ingredients. The interaction of the each component was investigated for its binding modes with different serum protein, thus implicating them as drug candidates in clinical settings. Overall, the study indicates that red wine has a composition of flavonoids, non-flavonoids, and phenolic acids that can interact with the amino acids in the key regions of the proteins to enhance their biological activity. Among them, rutin, resveratrol, and tannic acid showed good binding affinity. Therefore, the protein–ligand complexes provide the basis for further studies and are envisaged to possess beneficial properties that can enhance their role in clinical applications.

## Figures and Tables

**Table 1:** Bioactive substances and antioxidant activities of red wines x liter.

Indices	CSCarmel1	CSCarmel2	CSYarden1	CSYarden2
Polyph, mgGAE	2190.83 ± 9.43 ^a^	2230.73 ± 8.72 ^a^	1560.33 ± 6.32 ^b^	1610.42 ± 6.21 ^b^
Flavan, mgCE	241.84 ± 3.62 ^b^	253.94 ± 2.92 ^ab^	272.51 ± 4.33 ^a^	283.63 ± 3.73 ^a^
Flavon, mgCE	408.63 ± 3.63 ^a^	418.63 ± 5.11 ^a^	292.42 ± 2.54 ^b^	302.62 ± 5.24 ^ab^
Tannins, mgCE	152.54 ± 1.82 ^a^	156.24 ± 1.42 ^a^	51.33 ± 0.92 ^b^	52.43 ± 0.73 ^b^
Anthoc, mgCGE	137.53 ± 2.24 ^a^	140.23 ± 2.93 ^a^	97.91 ± 1.83 ^b^	101.22 ± 2.93 ^b^
ABTS, mMTE	19.84 ± 0.34 ^a^	20.45 ± 1.12 ^a^	13.94 ± 1.11 ^b^	14.85 ± 1.23 ^b^
FRAP, mMTE	5.84 ± 0.54 ^a^	6.18 ± 0.61 ^a^	4.12 ± 0.34 ^b^	4.46 ± 0.25 ^ab^
CUPRAC, mMTE	27.11 ± 1.14 ^a^	28.33 ± 1.65 ^a^	19.64 ± 1.65 ^b^	20.47 ± 1.76 ^b^
DPPH, mMTE	9.65 ± 0.87 ^a^	10.52 ± 1.12 ^a^	7.14 ± 0.65 ^b^	7.36 ± 0.73 ^b^
Rutin, mg	8.63 ± 0.54 ^a^	9.25 ± 0.87 ^a^	6.81 ± 0.56 ^b^	6.53 ± 0.55 ^b^
Resveratro, mg	2.15 ± 0.18 ^ab^	2.98 ± 0.12 ^a^	1.71 ± 0.17 ^b^	1.91 ± 0.17 ^ab^
Quercetin, mg	7.32 ± 0.41 ^ab^	8.24 ± 0.61 ^a^	5.74 ± 0.43 ^c^	6.49 ± 0.43 ^b^
Caffeic acid, mg	10.15 ± 0.97 ^a^	11.24 ± 1.12 ^a^	8.64 ± 0.76 ^b^	9.45 ± 0.75 ^ab^
Catechin, mg	40.21 ± 0.37 ^a^	42.17 ± 0.46 ^a^	31.18 ± 0.23 ^b^	34.15 ± 0.22 ^ab^
Epicatechin, mg	26.14 ± 2.33 ^a^	28.65 ± 2.43 ^a^	21.94 ± 2.09 ^b^	23.18 ± 1.89 ^ab^

Values are means ± SD of five measurements; *n* = 5 samples per vintage, each subsampled and analyzed five times. Means within rows with the different superscripts are statistically different (*p* < 0.05; Student’s *t*-test). Abbreviations: 1, 1-diphenyl-2-picrylhydrazyl (DPPH); Polyph, polyphenols; Flavon, flavonoids; Flavan, flavanols; GAE, gallic acid equivalent; CE, catechin equivalent; TE, trolox equivalent; Anthoc, anthocyanins; CGE, cyanidin-3-glucoside equivalent; ABTS, 2, 2-Azino-bis (3-ethyl-benzothiazoline-6-sulfonic acid) diammonium salt; FRAP, Ferric-reducing/antioxidant power; CUPRAC, Cupric reducing antioxidant capacity; CSCarmel1, CSCarmel2, Cabernet Sauvignon Carmel Selected, vintages 2017, 2019; CSYarden1 and CSYarden2, Cabernet Sauvignon Golan Heights Winery Yarden, vintages 2017 and 2019.

**Table 2:** Spectral data of the investigated samples and some standards.

Indices	CSCarmel 1	CSCarmel 2	CSYarden 1	CSYarden 2	Tannic Acid	Quercetin	Caffeic Acid	Ethanol	Fib/HSA
λ_em_/ex, nm, peak **a** Fib	230/341	230/341	230/340	230/341	229/343	228/340	229/340	228/341	229/342
FI, A.U., peak **a** Fib	468.8 ± 7.2 ^c^	478.4 ± 8.2 ^c^	484.6 ± 5.6 ^c^	499.6 ± 7.3 ^c^	657.4 ± 5.9 ^b^	861.0 ± 8.5 ^a^	836.1 ± 8.9 ^a^	865.5 ± 7.9 ^a^	883.6 ± 7.9 ^a^
BP, %, peak **a** Fib	46.9 ± 3.8^a^	45.9 ± 6.9 ^a^	45.2 ± 3.4 ^a^	43.5 ± 7.1 ^a^	26.6 ± 3.9 ^b^	2.6 ± 0.7 ^d^	5.4 ± 0.7 ^c^	2.1 ± 0.3 ^d^	-
λ_em_/ex, nm, peak **b** Fib	280/345	281/345	278/344	278/345	280/347	280/343	278/341	281/342	282/341
FI, A.U., peak **b** Fib	524.6 ± 9.3 ^c^	535.3 ± 6.9 ^c^	679.3 ± 5.3 ^bc^	702.3 ± 8.3 ^b^	595.9 ± 5.4 ^c^	706.9 ± 8.2 ^b^	768.7 ± 7.1 ^ab^	797.1 ± 7.8 ^ab^	811.7 ± 8.4 ^a^
BP, %, peak **b** Fib	35.4 ± 4.4 ^a^	34.1 ± 2.8 ^a^	16.3 ± 1.4 ^bc^	13.7 ± 1.2 ^bc^	26.6 ± 5.1 ^b^	12.9 ± 1.1 ^c^	5.3 ± 0.7 ^d^	1.8 ± 0.1 ^e^	-
λ_em_/ex, nm, peak **a** HSA	227/356	228/357	226/354	226/355	226/358	229/356	225/358	228/355	228/353
FI, A.U., peak **a** HSA	406.2 ± 7.3 ^c^	414.5 ± 7.2 ^c^	411.5 ± 4.3 ^c^	424.9 ± 3.1^c^	579.6 ± 4.7 ^b^	582.8 ± 5.1 ^b^	626.9 ± 7.4 ^a^	633.0 ± 9.1 ^a^	643.0 ± 6.3 ^a^
BP, %, peak **a** HSA	36.9+3.8 ^a^	35.5 ± 1.8 ^a^	36.0 ± 3.2 ^a^	34.0 ± 3.2 ^a^	9.9 ± 0.9 ^b^	9.4 ± 0.7 ^b^	2.5 ± 0.4 ^c^	1.6 ± 0.4 ^c^	-
λ_em_/ex, nm, peak **b** HSA	278/359	278/359	279/360	280/360	280/359	280/356	279/360	280/356	280/357
FI, A.U., peak **b** HSA	843.0 ± 6.4 ^ab^	860.2 ± 7.4 ^ab^	771.0 ± 7.4 ^b^	794.8 ± 5.2 ^b^	818.9 ± 9.9 ^ab^	867.4 ± 8.8 ^ab^	865.5 ± 7.9 ^ab^	910.9 ± 8.3 ^a^	920.1 ± 10.3 ^a^
BP, %, peak **b** HSA	8.4 ± 0.7 ^bc^	6.5 ± 0.8 ^c^	16.2 ± 1.5 ^a^	13.6 ± 1.9 ^ab^	11.0 ± 1.0 ^b^	5.7 ± 0.4 ^c^	5.9 ± 0.6 ^c^	1.0 ± 0.9 ^d^	-

The values are means ± SD of five measurements with *n* = 5 samples per vintage, and each was subsampled and analyzed five times. Means within rows with different superscripts are statistically different (*p* < 0.05; Student’s *t*-test). Abbreviations; CSCarmel1, CSCarmel2, Cabernet Sauvignon Carmel Selected, vintages 2017 and 2019; CSYarden1 and CSYarden2, Cabernet Sauvignon Golan Heights Winery Yarden, vintages 2017 and 2019; maximum emission/excitation peak (λ_em_/ex); fluorescence; intensity (FI); arbitral units (A.U.); fibrinogen (Fib); binding to HSA (%) and binding to Fib (%) are the percent decreases in fluorescence emissions of the fractions of the binding sites of the proteins occupied by the ligands.

**Table 3:** Binding affinity score of wine components docked with serum proteins.

Compound Name	PubChem ID	Binding Affinity (kcal/mol)			
		CRP	Fibrinogen	GPX3	HSA
Epicatechin	72276	−7.8	−5.1	−6.6	−8.9
Epigallocatechin	72277	−8.3	−6.3	−6.4	−8.6
Resveratrol	445154	−7.4	−6.1	−6.8	−9.1
Rutin	5280805	−8.7	−7.9	−7.4	−9.9
Quercetin	5280343	−8.7	−5.3	−6.8	−9.2
Gallic acid	370	−6.3	−5.7	−6.2	−6.2
Tannic acid	16129778	−7.7	−6.4	−7.3	−10.4
Myricetin	5281672	−8.4	−6.3	−6.8	−9
Caffeic acid	689043	−6.4	−5	−5.7	−7.2

## Data Availability

Not applicable.

## Sample Availability

Samples of the compounds are not available from the authors.
